# PENK inhibits osteosarcoma cell migration by activating the PI3K/Akt signaling pathway

**DOI:** 10.1186/s13018-020-01679-6

**Published:** 2020-04-25

**Authors:** Hai-ping Zhang, Zi-liang Yu, Bing-bing Wu, Fa-rui Sun

**Affiliations:** 1 Department of Orthopedics, Second Affiliated Hospital of Nantong University, Nantong, 226000 Jiangsu People’s Republic of China; 2 School of Medicine, Nantong University, Nantong, 226000 Jiangsu People’s Republic of China; 3 Department of Orthopedics, Huangshi Central Hospital, Affiliated Hospital of Hubei Polytechnic University, Edong Healthcare Group, Tianjin Rd, Huangshi, 435000 Hubei Province People’s Republic of China

**Keywords:** PENK, Osteosarcoma, PI3K, Akt, Migration

## Abstract

**Background:**

This article reports the effects of proenkephalin (PENK) on osteosarcoma (OS) cell migration.

**Methods:**

A Gene Expression Omnibus (GEO) dataset was used to identify differentially expressed genes (DEGs) in OS tumor samples and normal human osteoblasts. Tumor tissue and adjacent normal tissue were collected from 40 OS patients. MG63 cells were transfected with si-PENK. Transwell migration assays and wound healing assays were performed to compare the effect of PENK on migration. Moreover, LY294002 was used to identify the potential mechanism. Gene expression was examined via qRT-PCR and Western blotting.

**Results:**

Bioinformatic analysis revealed that PENK was downregulated in OS tumor samples compared with normal human osteoblasts. Moreover, PENK was identified as the hub gene of the DEGs. The PI3K/Akt signaling pathway was significantly enriched in the DEGs. Moreover, PENK was downregulated in OS and MG63 cells compared with the corresponding control cells. Silencing PENK promoted MG63 cell migration; however, treatment with LY294002 partially attenuated PENK silencing-induced OS cell migration.

**Conclusion:**

PENK inhibits OS cell migration by activating the PI3K/Akt signaling pathway.

## Introduction

Osteosarcoma (OS), a tumor of mesenchymal origin, is a common bone malignancy [1, 2]. OS is characterized by direct formation of osteoid tissue or immature bone by tumor cells [3] and accounts for approximately 60% of pediatric bone tumors [4]. Traditional treatment strategies for OS mainly include medical approaches such as multidrug chemotherapy and wide surgical excision [5]. However, the inhibitory effect of these strategies on the progression of OS is limited. The median survival time of OS patients is 13 months, and the 2-year and 5-year survival rates of these patients are 44% and 20%, respectively [6].

Recent advances in molecular genetic studies have improved our understanding of OS pathogenesis. Exploring the hub genes involved in OS could help identify therapeutic targets for OS. Previous studies identified FGF5 as an early diagnostic marker for OS [7]. In addition, Yang et al. [8] revealed that CCND1 expression was associated with poor prognosis of OS. However, no core gene that could be a therapeutic target for OS has been identified.

At the molecular level, multiple genes are differentially expressed during OS progression [9]. Proenkephalin (PENK) was originally shown to be expressed in the mature nervous and neuroendocrine systems through the opioid pathway and to be involved in the regulation of cell death and survival [10]. PENK has been identified as a key gene in multiple types of cancers [11–13].

However, the molecular mechanisms of PENK in OS remain elusive. In this study, the microarray dataset GSE12865 was downloaded from the Gene Expression Omnibus (GEO) repository, and bioinformatic analysis was performed. PENK was identified as a hub gene involved in the progression of OS. Moreover, our results suggested that PENK promotes OS cell migration by regulating the phosphatidylinositol 3-kinase (PI3K)/Akt signaling pathway.

## Materials and methods

### Differential gene analysis

The gene expression profile GSE12865, which is based on the Affymetrix Human Gene 1.0 ST Array platform, was downloaded from the GEO database (https://www.ncbi.nlm.nih.gov/geo/). The GSE12865 dataset contains 14 samples—12 OS tumor samples and 2 normal human osteoblast (HOB) samples. The limma R/Bioconductor software package was used in R (version 3.5.3) to identify the differentially expressed genes (DEGs) between the OS tumor samples and HOBs. The cutoff criteria were set as follows: |log fold change| > 2 and adjusted *P* value < 0.05. Volcano plots and heat maps for visualization of the DEGs were exported from R (version 3.5.3).

### Gene Ontology (GO) term and Kyoto Encyclopedia of Genes and Genomes (KEGG) pathway analyses

To explore the functions of significantly differentially expressed mRNAs and the corresponding signaling pathways, GO term and KEGG pathway enrichment analyses were conducted. GO term enrichment analysis was used to annotate the enrichment of the DEGs in gene functional terms in the molecular function (MF), biological process (BP), and cellular component (CC) categories. KEGG pathway enrichment analysis was used to identify signaling pathways enriched in the DEGs [14]. The clusterProfiler package was used to perform GO and KEGG enrichment analyses of DEGs [15]. *P* < 0.05 was considered to indicate statistical significance.

The Search Tool for the Retrieval of Interacting Genes (STRING) database (http://string-db.org/) was used to generate the protein-protein interaction (PPI) network dataset. A minimum required interaction score of ≥ 0.4 was selected as the cutoff value. The Molecular Complex Detection (MCODE) Cytoscape software plugin (version 3.7.1) was used to generate subclusters in the PPI network [16]. The advanced options were set as follows: degree cutoff = 2, node score cutoff = 0.2, and k-core = 2.

### Patients and tissues

A total of 40 pairs of OS tissue and matched adjacent normal tissue samples were obtained during surgery between January 2013 and January 2015. The inclusion criteria were as follows: (1) primary diagnosis of OS without any history of therapy, (2) no presence of other cancer-related diseases, (3) availability of data for final pathological diagnosis, and (4) no presence of systemic diseases, such as cardiovascular diseases. In addition, no restrictions were placed on the patients’ age or sex or on the anatomic site of the tumor. The patients’ information is summarized in Table 1. The patients did not differ significantly in age, sex ratio, tumor size, tumor anatomic location, serum level of lactate dehydrogenase, serum level of alkaline phosphatase, pathology, or metastasis status. However, a statistically significant difference in the clinical stage was found between patients with high expression of PENK and those with low expression of PENK (*P* < 0.05). Informed written consent forms were signed by all included patients, and the study protocol was approved by the Institutional Research Ethics Committee of Huangshi Central Hospital.

**Table 1:** Correlation of PENK expression with clinicopathological features of osteosarcoma

Clinicopathological features	Number of cases (*n*)	PENK expression		*P*
		High (*n*)	Low (*n*)	
Age				
< 25 years	28	16	12	0.573
≥ 25 years	12	8	4	
Gender				
Male	25	18	7	0.722
Female	15	10	5	
Tumor size				
> 8 cm	22	10	12	0.949
≤ 8 cm	18	8	10	
Anatomic location				
Tibia/femur	30	20	10	0.702
Elsewhere	10	6	4	
Serum level of lactate dehydrogenase				
Elevated	25	15	10	0.191
Normal	15	12	3	
Serum level of alkaline phosphatase				
Elevated	31	16	15	0.162
Normal	9	7	2	
Clinical stage				
IIA	32	25	7	0.004
IIB/III	8	2	6	
Pathology				
Osteoblastic	15	8	7	0.966
Chondroblastic	10	5	5	
Fibroblastic	10	6	4	
Telangiectatic	5	3	2	
Metastasis				
Absent	30	15	15	0.583
Present	10	6	4	

### Cell culture

Human OS cell lines (MG63, U2OS, 143B, and SAOS2) and an immortalized human fetal osteoblast cell line (hFOB1.19) were obtained from the American Type Culture Collection (ATCC, Manassas, VA, USA). All of these cells were maintained in RPMI-1640 medium containing 10% fetal bovine serum (FBS, Gibco; Thermo Fisher Scientific) at 37 °C in a humidified atmosphere of 5% CO_2_. The culture medium was replaced every 2 days. Confluent monolayers were split by treatment with phosphate-buffered saline (PBS) and 0.05% trypsin solution containing 0.05% ethylenediaminetetraacetic acid (EDTA) (Nanjing Jiancheng Biotechnology Institute). When the cell monolayer was between 70% and 80% confluent, the cells were treated with small interfering RNA (siRNA) negative control (si-con), si-PENK-1, and si-PENK-2.

### Transfection

Human OS cell lines and hFOB1.19 suspensions (1 × 10^4^ cells in 150 μL) were plated on coverslips in a confocal petri dish (NEST, Hong Kong, China) for transient transfection. DMEM (1 mL) containing 10% FBS was added to the dish, and the cells were cultured for 24 h prior to transfection. Transfection was performed using Lipofectamine 3000 (Thermo Fisher Scientific) and Opti-MEM reduced-serum medium (Life Technologies, Waltham, Massachusetts, USA) according to the manufacturer’s instructions.

siRNA sequences targeting PENK (si-PENK-1 and si-PENK-2) were purchased from GenePharma, Inc. The siRNA sequences were as follows: si-PENK-1 forward, 5′-GCAATCGAGATGGAACCAT-3′ and si-PENK-1 reverse, 5′-ATGGTTCCATCTCGATTGC-3′; si-PENK-2 forward, 5′-CCATCGGTCTACCTATTAT-3′ and si-PENK-2 reverse, 5′-TACGAACGACCAGCTTACC-3′; and si-con forward, 5′-UUCUCCGAACGUGUCACGUTT-3′ and si-NC reverse, 5′-ACGUGACACGUUCGGAGAATT-3′. LY294002 was purchased from MedChemExpress (MCE, Shanghai, China) and used at a concentration of 10 μM as previously described [17]. MG63 cells were pretreated with LY294002 (10 μM) and were then stimulated with si-PENK.

### Cell Counting Kit-8 (CCK-8) assay

The proliferation ability of the MG63 OS cell line was assessed using a CCK-8 assay (Dojindo, Japan). In brief, cells were plated in 96-well plates, and after incubation for 24 h, 48 h, and 96 h, CCK-8 solution was added. Then, the absorbance at 450 nm was measured with a spectrophotometer (Thermo Fisher Scientific).

### Cell migration assay

Cells were seeded in 6-well plates and grown to confluence. The cell monolayers were scratched with a micropipette tip and cultured in FBS-free medium. Photomicrographs were acquired at 0 and 24 h after wounding. Each wound healing assay was independently repeated three times.

### Migration assay

Transwell migration chambers (Corning Costar; pore size 8 mm) were used to assess the cell migration ability. A total of 5 × 10^4^ cells were suspended in 100 μL of FBS-free medium and added to the top chamber. The bottom chamber was filled with standard medium. Cells were incubated at 37 °C for 24 h. Cells that failed to migrate or invade were removed from the top surface of the membranes by wiping. Migrated and invaded cells on the bottom surface of the membranes were fixed and stained with 0.2% crystal violet. The migrated or invaded cells from five random areas of the membrane were counted using light microscopy. All experiments were independently performed three times.

### Reverse transcription PCR (RT-PCR)

Cells were extracted using TRIzol reagent (Invitrogen; Thermo Fisher Scientific). The concentration and purity of total RNA was determined by measuring the absorbance at 260 and 280 nm (A260/A280). Single-strand cDNA was synthesized by reverse transcription with a Superscript II Reverse Transcriptase Kit and oligo dT (12–18) primers (Invitrogen, Carlsbad, CA, USA) and amplified by RT-PCR with iQ SYBR Green Supermix (Bio-Rad, Hercules, CA, USA). Quantitative PCR (qPCR) was performed using SYBR Green PCR Master Mix (Applied Biosystems) following the manufacturer’s instructions. The expression levels of the target genes were calculated relative to that of the housekeeping gene GAPDH using the Stratagene Mx3000P software (Applied Biosystems) with the 2^−ΔΔCt^ method. The primer sequences used for PCR are shown in Table 2.

**Table 2:** Real-time PCR primers for amplification of specific MG63 OS cell line

Gene	Forward (5′-3′)	Reverse (5′-3′)
GAPDH	CTGGCACCACACCTTCTACA	GGTACGACCAGAGGCATACA
PENK	GCATGGCCAAGAAGACATCC	CCTCGGGTTTCCACGTCTC

### Western blotting (WB)

Total protein was extracted with RIPA buffer from cells and was then separated by sodium dodecyl sulfate-polyacrylamide gel electrophoresis (SDS-PAGE). The separated proteins were transferred to polyvinylidene difluoride membranes, which were blocked with 5% nonfat milk dissolved in Tris-buffered saline containing 0.05% Tween 20 (TBS-T) for 1 h at room temperature. Membranes were then incubated overnight at 4 °C with primary antibodies specific for PENK (Abcam, 1:2000), PI3K (CST 8486, 1:1000), Akt (Abcam ab8448, 1:1000), p-Akt (Abcam ab8564, 1:1000), and GAPDH (Proteintech, 1:8000). Finally, membranes were incubated for 1 h with horseradish peroxidase (HRP)-conjugated secondary antibodies (1:500) and visualized using an enhanced chemiluminescence system according to the manufacturer’s instructions.

### Statistical analysis

All statistical analyses were performed using the SPSS version 17.0 software. GraphPad Prism 7 was used to generate the graphs in this article. The data are expressed as the mean ± standard deviation (SD) values. Student’s *t* test or one-way analysis of variance was conducted to compare differences between groups. *P < 0.05 was considered to indicate a significant difference.

## Results

### Bioinformatic analysis results

**Fig. 1:**
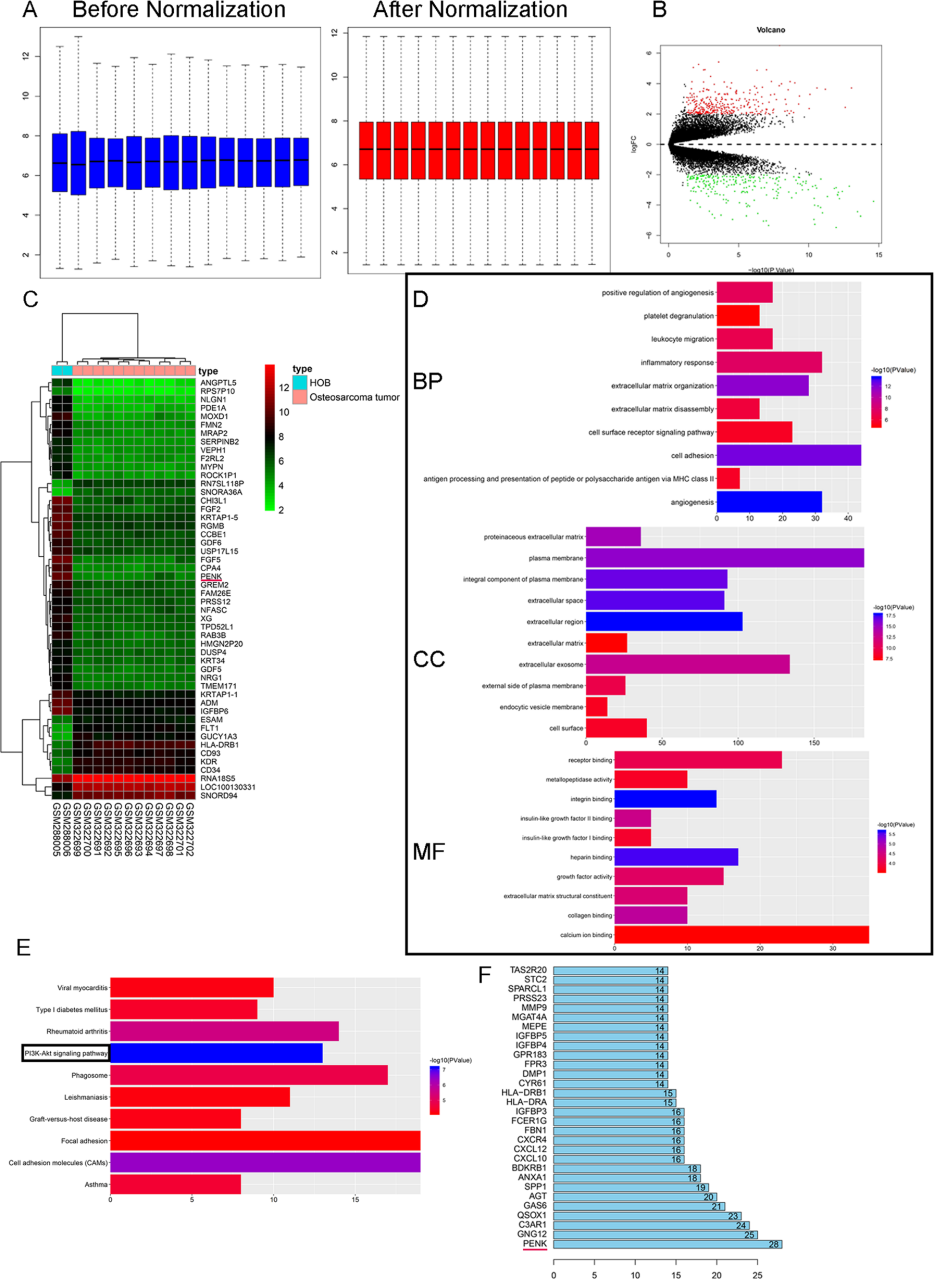
**a** Normalization of raw data from GSE12865. **b** Volcano plots of DEGs between OS tumor cells and HOBs. **c** Heatmaps of the top 50 DEGs from GSE12865. **d** Functional analysis of DEGs by BP, CC, and MF categories. **e** KEGG pathway analysis of DEGs. **f** Histogram of the top 30 genes with the number of edges

To decrease the fraction of false-positive findings, 14 sample values were log2 transformed and normalized by quantile normalization. The normalized data are shown in Fig. 1a. The volcano plots and the heatmap of the top 50 DEGs identified from GSE12865 are shown in Fig. 1b, c, respectively. A total of 568 DEGs were identified, among which 245 mRNAs were downregulated and 223 were upregulated. To explore the potential biological functions of the consensus genes, GO term analysis (Fig. 1d) and KEGG pathway analysis (Fig. 1e) were performed. The BP enrichment analysis results showed that the DEGs were significantly involved in extracellular matrix organization. The CC enrichment analysis results showed that the DEGs were significantly involved in the extracellular region. The MF enrichment analysis results showed that the DEGs were significantly involved in integrin binding. The KEGG pathway analysis results revealed that the PI3K/Akt signaling pathway was enriched in the DEGs. The PPI results showed that PENK exhibited the maximum binding score for interactions with other proteins (Fig. 1f).

### PENK is downregulated in OS and MG63 cells

**Fig. 2:**
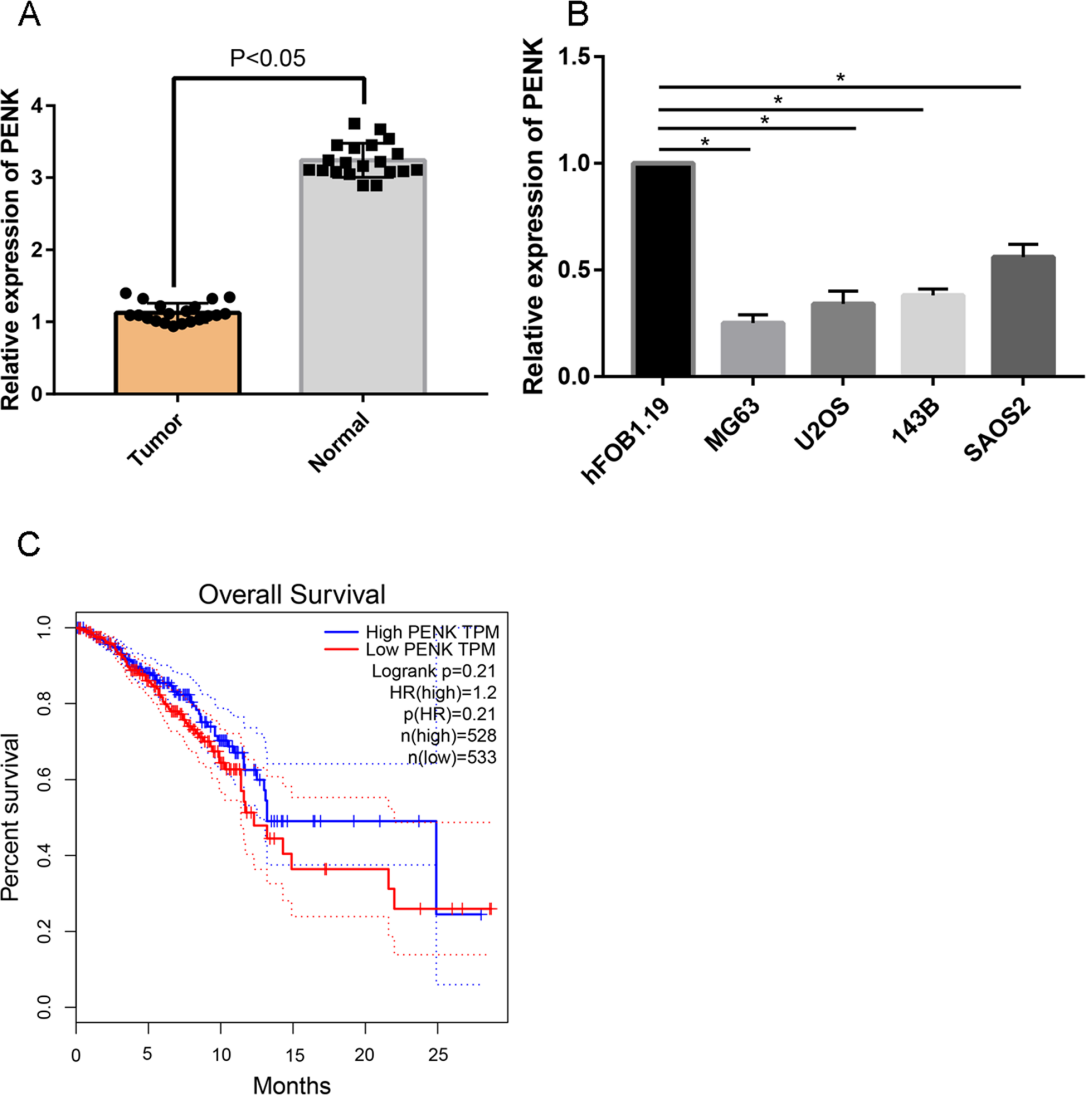
**a** Relative expression of PENK in tumors and normal tissues. **b** Relative expression of PENK in hFOB1.19 cells and human OS cell lines (MG63, U2OS, 143B, and SAOS2). **c** Overall survival analysis of OS patients with high and low expression levels of PENK. *P < 0.05

The qPCR results revealed that the relative expression of PENK was decreased in OS tumors compared with normal tissues (Fig. 2a). Moreover, downregulation of PENK gene expression was more obvious in MG63 cells than in the other cell lines examined. Therefore, MG63 cells were used for subsequent experiments (Fig. 2b).

The overall survival analysis results suggested that the overall survival of patients with high PENK expression was statistically significantly better than that of patients with low expression (Fig. 2c, *P* = 0.041).

### Silencing of PENK does not affect MG63 cell proliferation

**Fig. 3:**
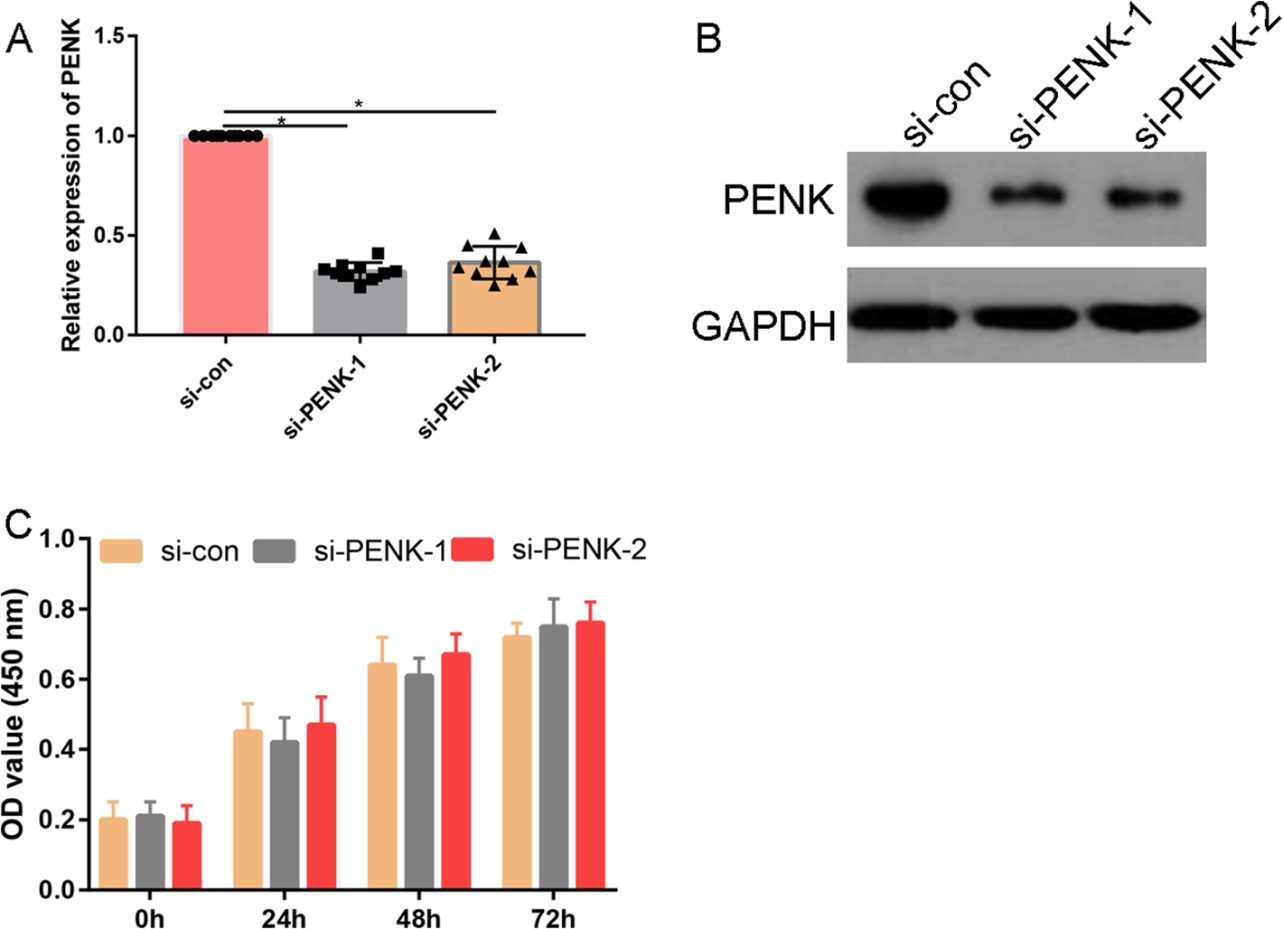
**a** Relative expression of PENK in si-con-, si-PENK-1-, and si-PENK-2-treated cells. **b** Western blot analysis of PENK expression in si-con-, si-PENK-1-, and si-PENK-2-treated cells. **c** Effects of si-PENK-1 and si-PENK-2 on MG63 cell proliferation, as assessed by a CCK-8 assay. *P < 0.05

Both the mRNA (Fig. 3a) and protein expressions (Fig. 3b) of PENK were appreciably downregulated after transfection with si-PENK-1 or si-PENK-2. The silencing efficiency was nearly 85%. We selected si-PENK-1 for the subsequent CCK-8 assay to determine whether si-PENK affects OS cancer cell proliferation. The CCK-8 assay results revealed that the number of OS cancer cells in all different treatment groups increased gradually. However, after 24, 48, and 72 h of treatment, the average number of viable cells did not differ significantly between the si-PENK-1- and si-PENK-2-treated groups and the si-con-treated group (Fig. 3c).

### Silencing of PENK promotes MG63 cell migration

**Fig. 4:**
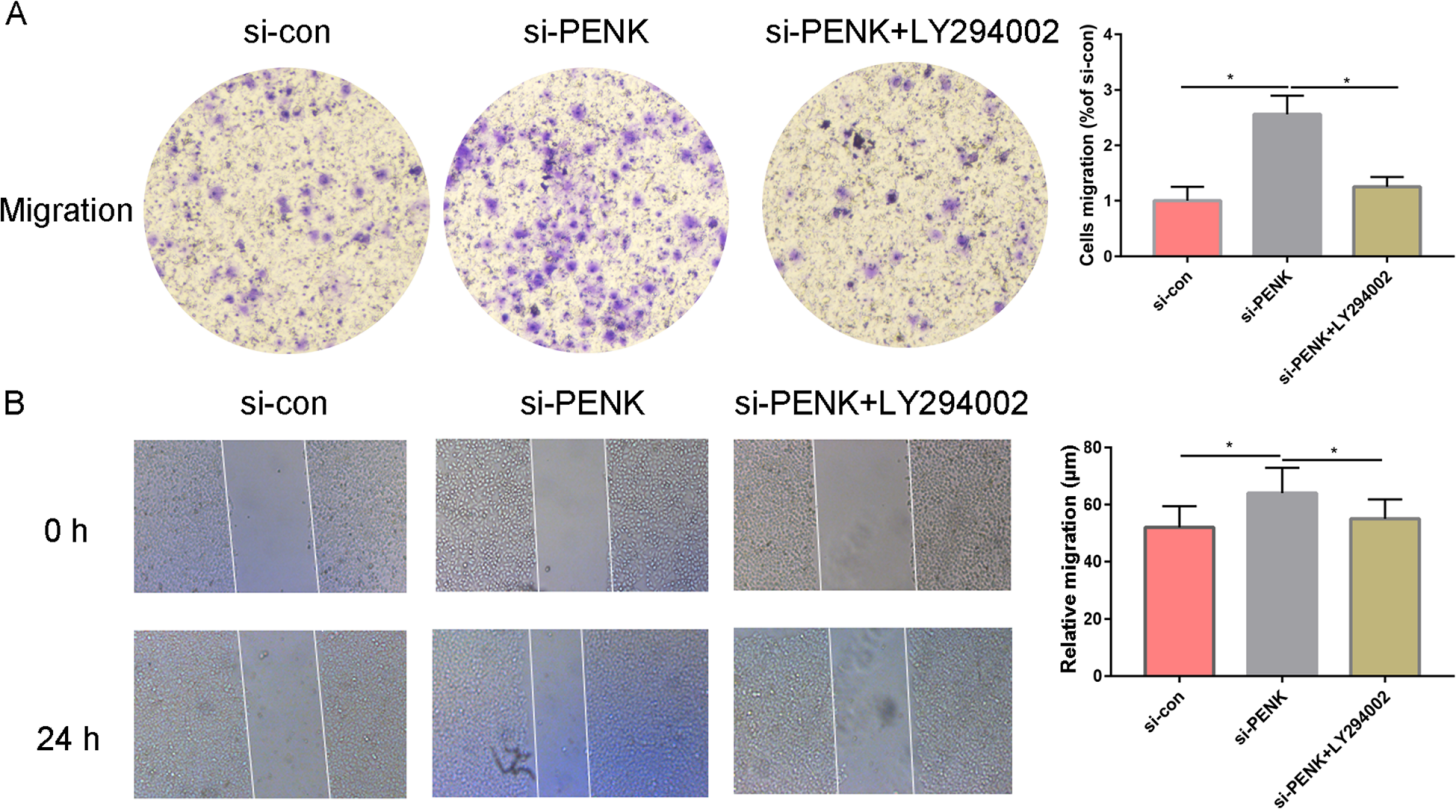
**a** A transwell assay was used to evaluate the invasion of MG63 cells after PENK downregulation. **b** A wound healing assay was used to evaluate the migration of MG63 cells after PENK downregulation. *P < 0.05

The migration ability of OS cancer cells was evaluated by a transwell migration assay and a wound healing assay. Compared with si-con treatment, si-PENK treatment significantly enhanced cell migration (Fig. 4a), as further confirmed by the wound healing assay (Fig. 4b). These results revealed that silencing PENK enhanced the migratory activity of OS cancer cells. Moreover, the si-PENK-promoted migration of MG63 cells was significantly blocked by LY294002 (Fig. 4a, b) compared with that of cells treated with si-PENK alone.

### Silencing of PENK promotes MG63 cell migration through the PI3K/Akt signaling pathway

**Fig. 5:**
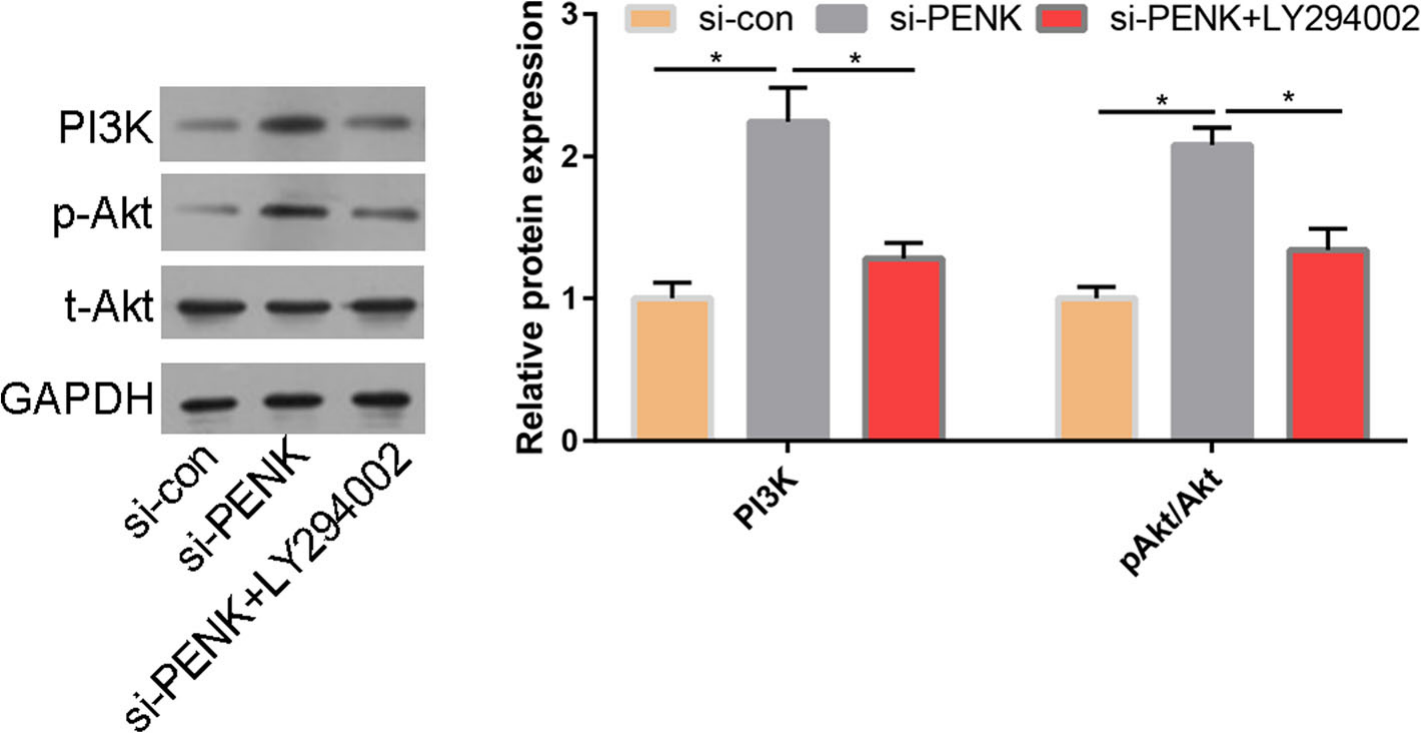
Silencing of PENK activated the PI3K/Akt pathway in MG63 cells. Protein levels of PI3K, total Akt (t-Akt), and p-Akt detected by WB in the si-con, si-PENK, and si-PENK+LY294002 groups

Next, we sought to determine whether silencing PENK affected PI3K/Akt pathway activity in MG63 cells. Silencing PENK significantly increased the phosphorylation of Akt and PI3K compared with that in si-con-treated cells (Fig. 5). Compared with silencing PENK alone, treatment with LY294002, a PI3K inhibitor, decreased the phosphorylation of Akt and the expression of PI3K in MG63 cells (Fig. 5).

## Discussion

OS cell migration plays a crucial role in OS progression, and a better understanding of the potential mechanism of OS will help reduce the number of patients with OS. Here, we provided the first evidence that PENK might inhibit OS migration by downregulating the PI3K/Akt signaling pathway.

In this study, we first used a public dataset (GSE12865) to assess the DEGs between HOBs and OS cells. PENK was identified as the hub gene involved in the progression of OS, and the PI3K/Akt signaling pathway was enriched in the DEGs. Further experiments validated that PENK expression was lower in human OS cells and tissues than in control cells and tissues. Moreover, we revealed that silencing PENK increased the migration of MG63 cells, and that this effect was mediated by the PI3K/Akt signaling pathway.

This study is the first to explore the expression and function of PENK in OS, specifically in human OS cells. PENK is expressed in several cancers and performs diverse biological functions. PENK and PENK-derived peptides are associated with the progression of head and neck cancer [18] and pancreatic cancer [19]. Tang et al. [20] found that PENK expression is closely associated with the prognosis of gastrointestinal stromal tumors, indicating that PENK may be a therapeutic target for gastrointestinal stromal tumors. Moreover, further studies conclusively showed aberrant methylation of PENK.

However, the role and potential mechanism of PENK in OS have not been comprehensively explained. First, analysis with the Bioconductor software package revealed that PENK was downregulated in OS tumor cells compared with HOBs. In addition, bioinformatic analysis further revealed that PENK was the hub gene involved in OS progression. Thus, we selected PENK for further evaluation.

The PCR and WB results further confirmed that PENK was downregulated in OS tumors. OS patients with high PENK expression had better overall survival than those with low expression. PENK, which is highly upregulated in the STA-ET-7.2 cell line, has been shown to promote stress-activated apoptosis through transcriptional repression of NF-kappaB- and p53-regulated gene targets. In addition, Aryee et al. [21] found that PENK was differentially expressed in Ewing sarcoma patients, and Rosen et al. [22] identified that PENK was expressed in nondifferentiated cells of diverse mesodermal lineages, and thus may play different roles in tumor development.

Next, siRNA was used to characterize downstream gene expression. Bioinformatic analysis revealed that PI3K was the most likely potential target of PENK.

The PI3K/Akt pathway is thought to be one of the most important oncogenic pathways in human cancers [23]. Migration of OS cells plays a crucial role in the progression of malignant tumors, and accumulating evidence indicates that the PI3K/Akt pathway promotes these aggressive behaviors. Musculoskeletal sarcomas, including OS, commonly develop aberrant activation of the PI3K/Akt signaling pathway [24]. Moreover, targeting the PI3K/Akt signaling pathway can be an approach for the treatment of sarcomas [25]. Zhang et al. [26] also found that inhibiting the PI3K/Akt pathway suppressed the progression of Ewing sarcoma. Here, LY294002, a PI3K inhibitor, was used to block the PI3K-Akt signaling pathway, and the si-PENK-promoted migration of MG63 cells was significantly blocked by LY294002. Taken together, these results indicate that PENK inhibits MG63 cell migration through the PI3K/Akt signaling pathway.

## Conclusion

In conclusion, we explored PENK expression in OS and found that PENK might inhibit OS migration by downregulating the PI3K/Akt signaling pathway. Therefore, upregulation of PENK may be a promising treatment approach for OS.

## Data Availability

We declare that the materials described in the manuscript will be freely available to all scientists for noncommercial purposes.

## Abbreviations

PENK: Proenkephalin
OS: Osteosarcoma
GEO: Gene Expression Omnibus
MDA: Malondialdehyde
HOBs: Human osteoblasts
DEGs: Differentially expressed genes
GO: Gene Ontology
KEGG: Kyoto Encyclopedia of Genes and Genomes
BP: Biological process
MF: Molescular function
CC: Cellular component
STRING: Search Tool for the Retrieval of Interacting Genes
MCODE: Molecular Complex Detection
PBS: Phosphate-buffered saline
EDTA: Ethylenediaminetetraacetic acid
SDS-PAGE: Sodium dodecyl sulfate-polyacrylamide gel electrophoresis
TBS-T: Tris-buffered saline with 0.05% Tween 20
HRP: Horseradish peroxidase
SD: Standard deviation
